# Sex Differences in Group Composition and Habitat Use of Iberian Free-Range Pigs

**DOI:** 10.3389/fvets.2020.600259

**Published:** 2020-12-03

**Authors:** Antoni Dalmau, Míriam Martínez-Macipe, Xavier Manteca, Eva Mainau

**Affiliations:** ^1^Institute of Agrifood Research and Technology (IRTA), Animal Welfare Program, Girona, Spain; ^2^Universitat Autònoma de Barcelona (UAB), Veterinary School, Campus Universitat Autònoma de Barcelona, Cerdanyola del Vallès, Spain

**Keywords:** behavior, habitat, Iberian, outdoor, pig, population structure, sex differences

## Abstract

The aim of the present work was to study group size, group composition and habitat use of Iberian pigs along the year when reared outdoor. This consists of a regimen in which animals are reared free range from 2 months of age until at least 14 months of age. In a first stage, animals are supplemented with concentrates, and in a second, called montanera, pigs eat just natural resources in areas with no more than two pigs per hectare. In these systems, males are castrated to avoid boar taint and females spayed to avoid the attraction and mounting by wild boars. The study was carried out in five different farms allocated in the south-west of Spain during 2 consecutive years, from March 2012 to February 2014, under the montanera regimen, and with a total of 995 animals observed (498 males and 497 females). The data were analyzed with SAS by means of general models and proc mixed. Mean group size along the year was of 17 ± 12.9 individuals, but this was significantly lower (*P* < 0.05) during the montanera (12 ± 0.8) and at midday (13 ± 0.8). Groups were bigger (*P* < 0.05) when they were more than 50 m from a tree (23 ± 1.8), or <10 m from the shelter (25 ± 1.5), the feeding area (31 ± 3.1) and the water-bath area (25 ± 1.5). Nine percent of the groups were solitary animals, being higher (*P* = 0.0286) during the montanera (11%) than the rest of the year (8%) and being formed in 68% by males. Males were less involved in mixed groups than were females (75% vs. 91%), especially in spring, where the largest (*P* < 0.0001) male groups were found. Female groups were less frequent and smaller (*P* < 0.0001) than were male and mixed groups. In conclusion, although males were castrated at a very young age, they showed a different behavior than females, forming in bachelor groups during the spring and being less involved in mixed groups and with more solitary animals. During the montanera, when animals were feeding on acorns and other natural resources, groups were smaller and closer to the trees, solitary males reaching a maximum percent.

## Introduction

The Iberian Pig is a native breed of the Iberian Peninsula that originates from *Sus scrofa mediterraneous* and is reared in the south-west of the Iberian Peninsula (1). The Iberian Pig is characterized by its fat-production ability (2) and high rusticity. This enables the animals to cope better with climatic hazards. The Iberian Pig has been raised for centuries to produce dry-cured meat. Currently, the meat is considered “Iberian” when it comes from a pig with a minimum of 50% of Iberian genetics, where the mother must be 100% Iberian (RD 4/2014). In the most traditional production system, Iberian piglets are weaned to at least 2 months old, and then they are mixed in large pastures. In these areas, they are given concentrate at the same time as having natural resources for several months, until reaching around 90 kg−115 kg of body weight (2). The whole reproductive cycle of the animal is planned to provide an adequate physiological status capable of taking advantage of La Dehesa during the late fattening phase. La Dehesa is a Mediterranean ecosystem based on oak forest (*Quercus rotundifolia* and *Quercus suber*) with herbaceous species, mainly grasses and legumes, where animals can graze (3). In fact, in late autumn, when the acorns from the oaks (*Quercus ilex*) and cork oaks (*Quercus suber*) fall, pigs eat only the acorns and other natural products like tubers, grasses, legumes, fungi or roots from the dehesa pastures (4). This late fattening phase at La Dehesa is called “montanera” and the pigs must gain their last 50 kg−60 kg based only on that type of food source (5). In fact, pigs feeding freely during the montanera season are regarded as a distinctive mark of the best quality Iberian pig products (6). Rodríguez-Estévez et al. (7) measured a daily consumption of 7.1 kg−8.4 kg of acorns and 2.0 kg−2.7 kg of fresh grass during this phase. Iberian pigs should be slaughtered at least at 14 months of age and a body weight of 150 kg−160 kg (RD 4/2014). The regulation requires <2 pigs/ha during the montanera; however, the stocking rate used is lower in practice, because pigs are not allowed to receive any kind of feed or alimentary supplement during this phase. Hence, the stocking rate is typically <1 pig/ha (8).

Pigs are social animals and usually live in groups (9, 10), performing a wide repertoire of sexual, feeding and social behaviors (11, 12). Living in groups is regarded as an anti-predator tool for many ungulates: it reduces the predation risk, on the one hand, by the numerical dilution and, on the other, by having more vigilant animals able to warn the others about a coming danger. This anti-predator adaptation is especially common in species living in open habitats (11) and it is part of a cooperative behavior in which some individuals may benefit from having others entering potentially dangerous areas first (13). However, pigs could find more protection in areas covered by trees, where group sizes could be smaller. It is assumed that domestic pigs, if given the chance, show the same behavior repertoire as do wild boars (9, 14). In fact, Stolba and Wood-Gush (1989) described wild boars as gregarious animals that typically live in groups, mainly composed of females with their offspring, in matriarchal hierarchies, and Copado et al. (15) observed in free-ranging domestic pigs of different ages that adult females were the group leaders with strong cohesion and stable social hierarchies. This sexual segregation has been observed in other ungulates and determines different group sizes and habitat uses in males and females (16). In fact, space preferences for different activities have been described in pigs (17, 18).

The traditional Iberian pig production system provides an opportunity to study the flexibility of social organization of a large group of pigs at an extremely low stocking rate. However, two considerations should be considered. The first one is that in contrast to natural wild boar or even feral pigs, in these groups all animals have a similar age. The second one is related with the reproductive status. Although there is limited information available on the precise time of sexual maturity in Iberian pigs, it has been described that the Iberian female pig begins puberty at an average age of 6 to 7 months (3, 19), and for boars it has been described to be from 7 to 9 months of age (20). Therefore, males and females are reared for several months after sexual maturity. As a result, in males, castration is required to avoid boar taint in their final product (21), and females are spayed. The negative effect on growth due to oestrous behavior in Iberian females (22, 23) or in other breeds slaughtered at heavy weights (24) has been suggested to justify the need for spaying, but according to Dalmau et al. (25), the main reason to justify spaying in these females is the presence of wild boars in La Dehesa. In fact, the cohabiting of wild boars with entire females can produce a high percentage of pregnancies, which disturb the production cycle and could have sanitary and ethical problems.

Rodríguez-Estévez et al. (1), studied group size and resting locations of free-range pigs during the montanera and concluded that pigs split into small groups during daytime to forage but then rest together in a larger group to spend the nights in a common area. Nevertheless, no studies exist regarding the group size or composition (male, female, and mixed groups) in these animals along the year. This should include the two types of feeding schedules: first, when they are fed with concentrate given by the farmer, and later, during the montanera, when they need to search for food available only from natural resources. This changes the activity budgets (26), so it is expected to produce changes in the group size and composition. Finally, in swine species the dominance order is resource-related (27), so it is hypothesized that different kinds of resources in free-range situations, such as trees, shelter, water bath area, drinking or feeding area and fences could interact with group size and composition in Iberian pigs reared outdoors.

Accordingly, the aim of the present study was to study the group size and composition (males, females and mixed groups) in relation to their feeding regimen (animals fed with concentrates or maintained at montanera with no supplementation), and distance to different resources (trees, shelter, feeding area, drinker, fences and water bath area) along the year in Iberian pigs reared in outdoor conditions in five different farms in the south-west of Spain.

## Materials and Methods

Data were collected at five different farms dedicated to Iberian pig production in Extremadura, Spain. The study was performed during two consecutive production cycles (from spring, at 3 to 6 months of age, until slaughter the following winter, at 14 to 17 months age), from 2012 to 2014. In consequence, each pig was studied for around 12 months. Pigs were in all cases pure Iberian breed or crossed to 50% with duroc (male duroc x female Iberian pig). Animals 100% Iberian were born around September the previous year, and animals 50% Iberian were born around December the previous year. A farm contained only one type of genetics. The quantity of animals studied varied with the farm and production cycle (Table 1). In all farms, males and females were neutered according to the commercial practices in the area. In the case of males, this means that surgical castration was performed before 1 week of age, and in the case of females that spaying was performed at around 2 months of age. Sex ratios of female:male ranged from 0.8 to 1.3. After weaning, at around 2 months of age, pigs were allocated in the study areas, between 1 and 3 months before beginning the observations in March, and they were never mixed again with unknown animals. From this moment, pigs were reared in a free-range area where a zone to receive food (feeding area), drinkers, a shelter and a pond (water bath area) were provided within a total space allowance from 7 to 67 pigs per hectare. The free-range area included oaks and other normal dehesa vegetation, and pigs were fed with concentrates (3,000 Kcal EM/kg) once a day, in the morning, in a restricted regimen that ranged from 2 kg per animal during the spring and early summer to 1 kg per animal in the most restricted period at the late summer and early autumn. When the acorns started to fall in early winter, the montanera period started (Table 1), and pigs (1) were moved to a bigger landscape or (2) another part of the free-range area was open to provide more space and resources, in all cases ensuring a density of less than two pigs per hectare, with a maximum study area of 180 ha. During this period, animals were not fed by farmers.

**Table 1:** Number of visits to each farm, percentage of purebred, quantity of pigs in the 1st and 2nd year, available area during the year and during montanera, dates of montanera start for the 1st and 2nd year of study.

	**Farm 1**	**Farm 2**	**Farm 3**	**Farm 4**	**Farm 5**
Visits	28	44	15	26	17
% Purebreed	50%	100%	100%	100%	50%
Pigs at first year	94	127	145	140	141
Sex ratio (females/males)	0.9	1.1	1.3	1.1	0.8
Space allowance during the no montanera	13 pigs/hectare	11 pigs/hectare	18 pigs/hectare	47 pigs/hectare	14 pigs/hectare
Pigs at second year	61	87	0	200	0
Sex ratio (females/males)	1.3	0.9	0	0.9	0
Space allowance during the no montanera	8 pigs/hectare	7 pigs/hectare	0	67 pigs/hectare	0
Date to the first montanera	13/11/2012	21/11/2012	23/11/2012	14/12/2012	12/11/2012
Space allowance during the montanera	1.8 pigs/hectare	0.7 pigs/hectare	1.8 pigs/hectare	1.2 pigs/hectare	1.8 pigs/hectare
Date to the second montanera	14/11/2013	19/11/2013	0	6/11/2013	0
Space allowance during the montanera	1.2 pigs/hectare	0.5 pigs/hectare	0	1.6 pigs/hectare	0
Landscape characteristics	Mountainous area (slopes >30°)	Plain	Plain	Small hills	Small hills

Each farm was visited once every 1 or 2 weeks, from 7:30 to 21:00 h. Eight percent of the observations were carried out from 07:30 to 09:30 h, 56% from 09:30 to 11:30 h, 27% from 11:30 to 13:30 h, 2% from 13:30 to 15:00 h and 7% from 17:00 to 21:00 h, avoiding the times when animals were fed during the non-montanera period. To observe the animals, the same observer traveled every day to different farms (allocated a mean time of 1 to 2 h from each other). The animals were approached on foot and observed from a distance, trying not to be noticed by using binoculars. During a pilot study for the selection of farms, the different resources found in the study area were defined and described, such as trees, water bath area, shelter, feeding area and fences, which are common elements in this type of farms. The feeding area consisted in a concrete zone with 0.5 to 1.0 m^2^ per animal where pigs were fed all at the same time by leaving the concentrate on the floor. This area was just besides the entry to the landscape for facilitating the storage and disposal of feed. Drinkers were in all cases allocated besides the feeding area too. Only in farm 4 the shelter was at < 300 m to the feeding area (around 220 to 260 m) and in all the rest of cases it was at more than 300 m of distance. The water bath area was as well in all cases allocated to more than 300 m from the feeding and shelter areas, except in farm 4 that it was at 250 m from the shelter. During the visits, the observer indicated, for each group of animals, how far the group was from each element (<10 m, between 10 and 50 m or >50 m); how many animals and, when possible, what gender (male or female), formed the group. A group was considered any animal or animals located at more than 50 meters from any other animal (9, 28). A Leica Disto (Barcelona, Spain) distance-meter was used for the distance measurements. For each visit, two observation blocks were made 2 h apart. A total of 260 observations were performed. The assessments were performed from a distance to ensure that animals were followed when walking, foraging or lying. The four seasons of the year were considered as well: spring (March, April and May, with 18% of the visits), summer (June, July and August; with 38% of the visits), autumn (September, October, November; with 23% of the visits) and winter (December, January and February, with 21% of the visits). The montanera could be slightly different among different farms, but it covered mainly the late autumn and all winter (Table 1). Three periods of the day were considered for the analysis, morning (from 7:00 to 11:59 h), midday (from 12:00 to 14:59 h) and afternoon (from 15:00 to 21:00 h). Therefore, in the present study, the term montanera includes a specific period of the year (late autumn and winter), when the animals were older, had more space (at least one hectare per two pigs) and were fed just with natural resources (with a high presence of acorns). In addition, the season/age effect was linked to an age effect, was animals were younger in spring and older in winter.

### Statistical Analysis

Analyses were carried out with the Statistical Analysis System (SAS software, SAS Institute Inc.; Cary, NC, USA). The size of the observed groups (1,247 in total) were studied according to the fixed effects: montanera (yes or no), time of the day (morning, midday and afternoon), season/age (spring, summer, autumn and winter), and distances to fences, trees, water bath areas, shelter, feeding area and drinkers (<10, 10–50, and >50 meters) and interactions. Space allowance was as well-included as covariable. For those groups in which all males and females were identified (579 in total), the models considered as a fixed effect the same as that described previously, considering as well the type of group (only females, only males and mixed groups) and interactions between type of groups and the rest of fixed effects. The observation day and the farm*year effect (1 to 8 herds taking into account the five and three farms assessed the first and second year, respectively) were also included in the models as a repeated measure. As residuals were normally distributed, mixed models by means of Proc Mixed were used. The least-square means of fixed effects (LSMEANS) was used when the analysis of variance indicated differences. For the analysis of solitary animals or presence of males and females in mixed groups a proc Glimmix with a binomial distribution was used. For the incidence of different group types (male, female and mixed groups) a proc Glimmix with a multinomial distribution was used. In addition, spearman correlations were studied for the distances to different resources in relation to the season/age of the animals (from spring to winter), montanera and moment of the day (from morning to afternoon). In all cases, significance was fixed at *P* < 0.05.

## Results

### Group Size

**Figure 1:**
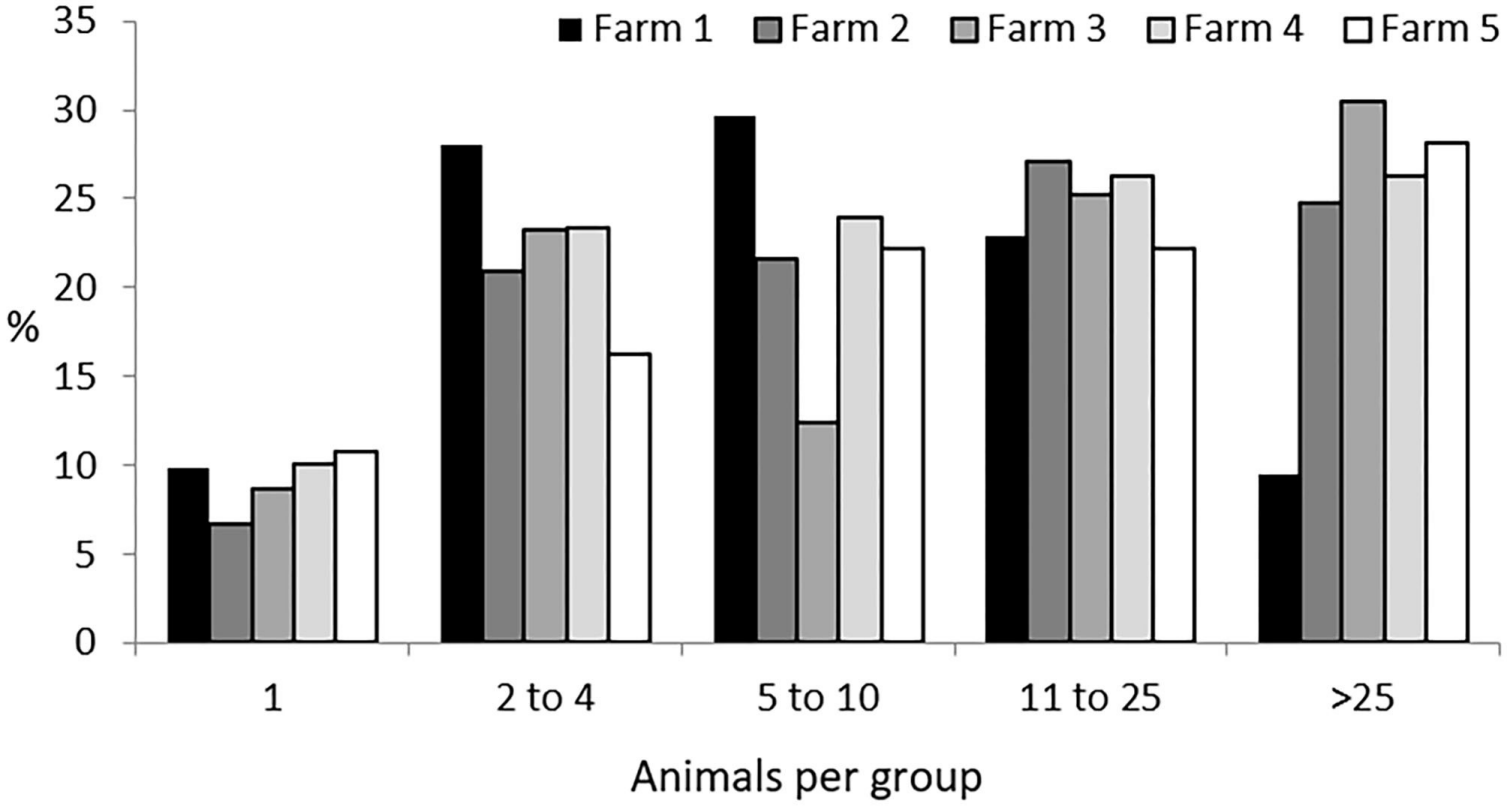
Percentage of groups of different sizes (one; two to four; five to 10; 11 to 25; and more than 25 animals per group) per each farm (1 to 5) for Iberian pigs reared free-range from a total of 1,247 groups observed.

The five herds studied the first year and the three studied the second, accounting for a total of 647 and 348 Iberian pigs, respectively, were found to have formed a total of 1,247 groups. This represents five groups per observation period, with a mean ± SD size of 17 ± 12.9 individuals per group. Solitary animals, those found more than 50 meters apart from any other individual, represented 9% of the groups. A group containing all the animals of a herd was only seen in seven observations: in Farm 2 four times, with 127 individuals; in Farm 4 just once, with 200 individuals, and in Farm 5 twice, with 141 individuals. In all of these cases, this occurred during the non-montanera period. The most typical size of groups was from two to 25 animals (two to four animals accounting for 23% of the total; five to 10 animals, 24% of the total, and 11 to 25 animals, 25% of the total). Finally, groups larger than 26 animals accounted for a total of 19% of the total observations. Considering all groups observed, those found in Farm 1 represented 20% of all groups observed, those from Farm 2, 31%, those from Farm 3, 10%, those from Farm 4, 27%, and those from Farm 5, 12% (Figure 1).

**Figure 2:**
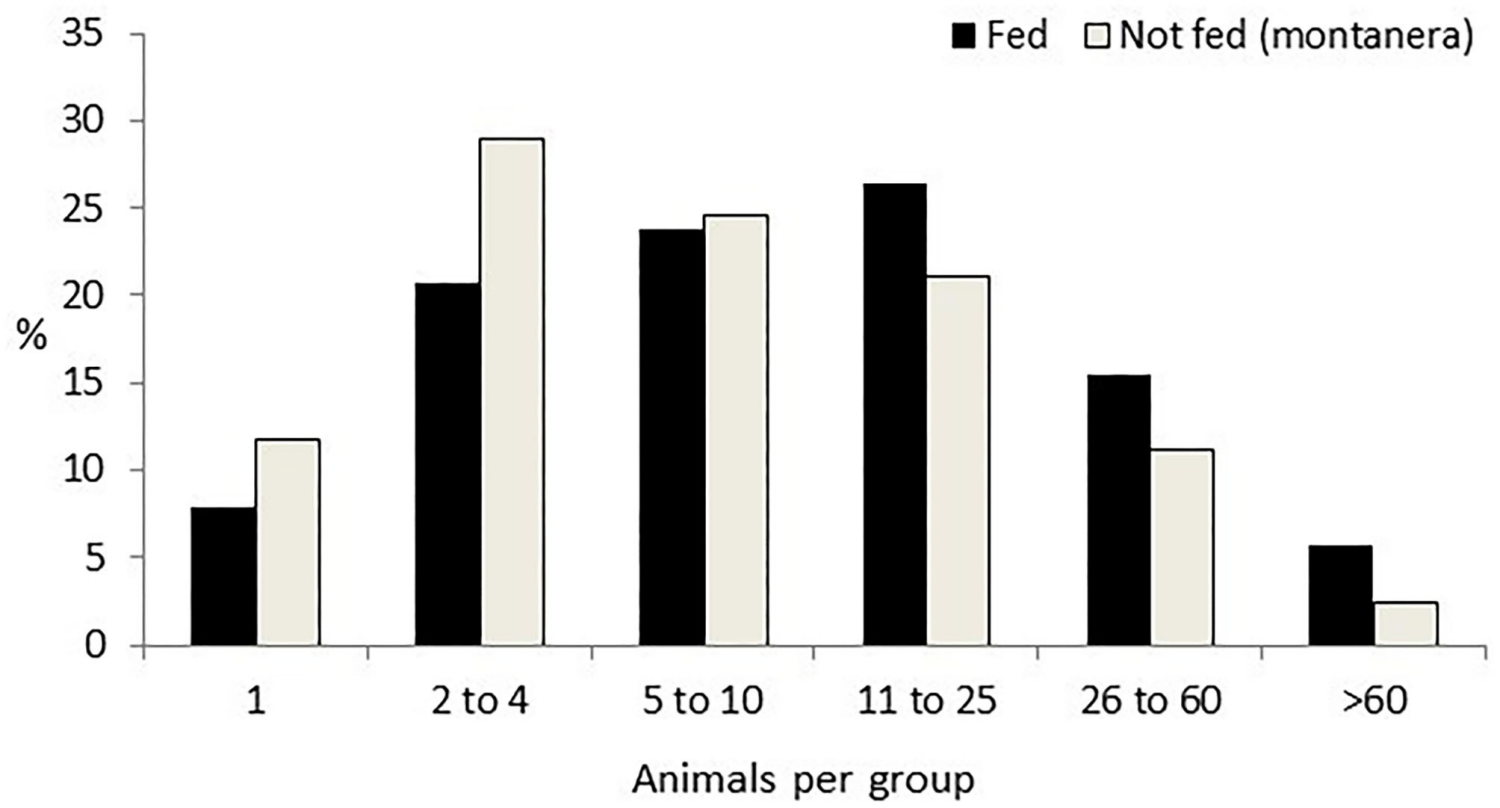
Percentage of groups of different sizes (one; two to four; five to 10; 11 to 25; 26 to 60; and more than 60 animals per group) in Iberian pigs reared free-range when being fed with concentrates (Fed) and when feeding just with natural resources [Not fed (montanera)] from a total of 1,247 groups observed.

Group size was affected by the montanera (*P* = 0.0008), by the time of the day (*P* = 0.0044), distance to the trees (*P* = 0.0264), distance to the feeder (*P* < 0.0001), distance to the water bath area (*P* = 0.0199), and distance to the shelter (*P* = 0.0084), with just a trend for season (*P* = 0.0974). Twenty-nine percent of the groups were observed during the montanera period, being smaller (12 ± 0.8 individuals per group) than the other 71% of the groups found the rest of the year (19 ± 0.8 individuals per group; Figure 2). Groups were smaller at midday (mean size ± SE; 13 ± 0.8 individuals per group), as compared with the morning (19 ± 1.0 individuals per group) or the afternoon (19 ± 2.4 individuals per group). Group size at more than 50 m from a tree was bigger (23 ± 1.8 individuals per group) than at 10 to 50 m (16 ± 1.4 individuals per group) or <10 m from a tree (18 ± 1.4 individuals per group). Group size at <10 m from a feeder was bigger (31 ± 3.1 individuals per group) than at 10 to 50 m (13 ± 2.8 individuals per group) or more than 50 m from the feeder (14 ± 1.7 individuals per group). Group size at <10 m from the water bath area was bigger (25 ± 2.9 individuals per group) than at more than 50 m from a water bath area (19 ± 2.3 individuals per group). Finally, group size at <10 m from a shelter was bigger (25 ± 1.5 individuals per group) than at 10 to 50 m (15 ± 1.2 individuals per group) or more than 50 m from the shelter (18 ± 1.4 individuals per group).

Globally, the 1,247 groups were closer to the trees (66% of the groups being at a distance of <10 meters from a tree) than to the feeding area, drinker, water bath area, shelter and fences (Table 2). The second structure with more groups situated to <10 meters away was fences, with 38% of the groups seen at this distance. On the other hand, most of the groups were seen to be more than 50 meters from shelters, drinkers, water bath and feeding area (90, 89, 83, and 76%, respectively). To be closer to a tree was positively correlated with the montanera (*r* = 0.40) and season/age of the animals (*r* = 0.50, Table 2). Groups of solitary animals represented 7% of the total in spring, 8% in summer, 12% in autumn and 10% in winter. However, no significant differences were found among seasons for this type of group. In contrast, the percentage that groups of solitary animals represented of the total was higher (*P* = 0.0286) during the montanera period (11%) than the rest of the year (8%).

**Table 2:** Spearman correlation results for the effects montanera (being fed just with natural resources, with a maximum density of 2 pigs per hectare in the late autumn and winter) in comparison to no montanera (being fed with natural resources and concentrate, with a maximum density of 67 pigs per hectare in spring to early autumn), later during the day (defined in base to observations during the afternoon and midday in comparison to the observations in the morning), and older (related with the age of the animals along the year, being the oldest in winter and the youngest in spring) according to distances to trees, drinker, feeder, water bath area, fences, and shelter areas (positive correlation showing a shorter distance to these resources and negative correlation a longer distance).

		**Montanera**			**Moment of the day**				**Season**				
		**Yes (%)**	**No (%)**	***r***	**Morning (%)**	**Midday (%)**	**Afternoon (%)**	***r***	**Spring (%)**	**Summer (%)**	**Autumn (%)**	**Winter (%)**	***r***
Distance to trees	<20 m	95	53	0.40	63	74	41	0.06	27	59	84	94	0.50
	20–50 m	3	9		6	8	14		10	10	2	4	
	>100 m	2	38		31	18	45		63	31	14	2	
Distance to Feeder	<20 m	4	16	−0.21	15	7	16	−0.12	13	16	17	2	−0.13
	20–50 m	6	13		13	8	11		9	15	13	4	
	>100 m	90	71		72	85	73		78	69	70	94	
Distance to drinker	<20 m	2	8	−0.14	7	4	13	NS^*^	7	9	6	2	−0.08
	20–50 m	2	5		4	3	13		4	5	7	2	
	>100 m	96	87		89	93	74		89	86	17	96	
Distance to water bath	<20 m	4	11	−0.14	8	9	20	NS	5	14	10	4	NS
	20–50 m	5	9		8	9	4		6	10	9	5	
	>100 m	91	80		84	82	76		89	76	81	91	
Distance to fences	<20 m	31	40	−0.09	42	30	39	−0.10	34	41	39	32	NS
	20–50 m	16	15		15	15	13		18	12	15	17	
	>100 m	53	45		43	55	48		48	47	46	51	
Distance to shelter	<20 m	2	9	−0.14	7	6	11	NS	18	5	5	2	−0.17
	20–50 m	1	3		3	2	9		2	4	3	1	
	>100 m	97	88		90	92	80		80	91	92	97	

* *NS, not significant correlation at P < 0.05*.

### Type and Composition of the Groups

Considering the 579 groups where all the animals could be identified by sex (male or female), 14% were formed only by females, 26% only by males and 60% were mixed groups (with at least one male or one female). An effect of season/age (*P* = 0.0147) was found for group composition, autumn, with the lowest percentage of mixed groups (15, 33, and 52% of female, male and mixed groups, respectively), being statistically different from summer (12, 23, and 65% of female, male and mixed groups, respectively) and winter (16, 21, and 63% of female, male and mixed groups, respectively). In addition, spring (15, 25, and 61% of female, male and mixed groups, respectively) was different from summer.

**Figure 3:**
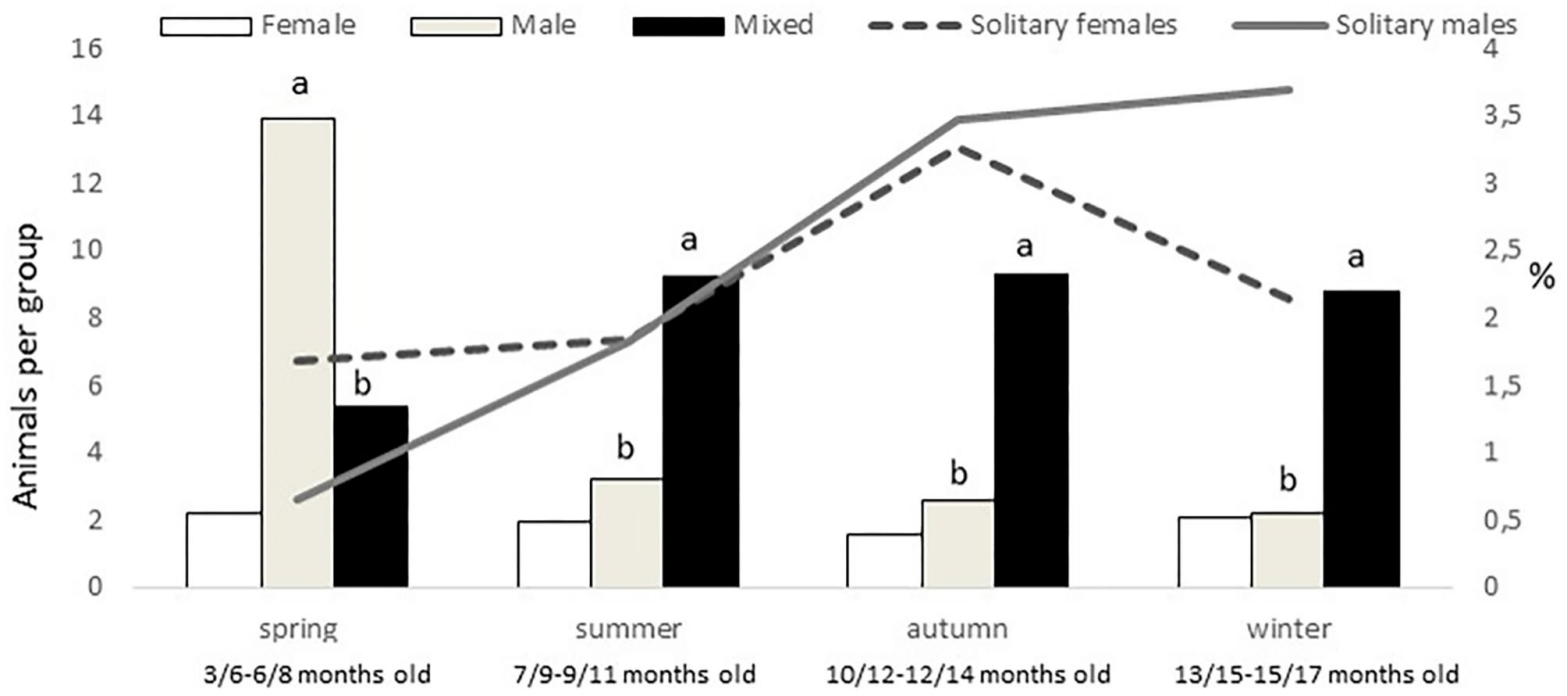
In the left axis and with bars, mean number of animals per group (female, male, and mixed groups). In the right axis and with lines, percentage of solitary females and males out of the total females and males observed for the different seasons (spring: March to May; summer: June to August; autumn: September to November and winter: December to February) in free-range Iberian pigs, being the youngest in spring and the oldest in winter.

An effect of group type was found on group size (*P* < 0.0001), female groups being smaller (mean 5 ± 0.9 animals per group) than male and mixed groups (9 ± 0.8 and 11 ± 1.3 animals per group, respectively). In addition, a montanera effect (*P* = 0.0020), an interaction montanera*group type (*P* = 0.0004), and an interaction season*group type (*P* < 0.0001), but not an effect of season (*P* = 0.1833) were found for group size. While female and mixed group sizes were not different between montanera and no montanera periods, male group size was higher (*P* < 0.0001) during the no montanera (4 ± 1.3 individuals per group) than during the montanera (2 ± 0.2 individuals per group). Actually, male groups were bigger in spring to any other group type in this season and to the male group size at any other season (Figure 3), while mixed groups at spring were smaller than at any other season/age (Figure 3).

**Figure 4:**
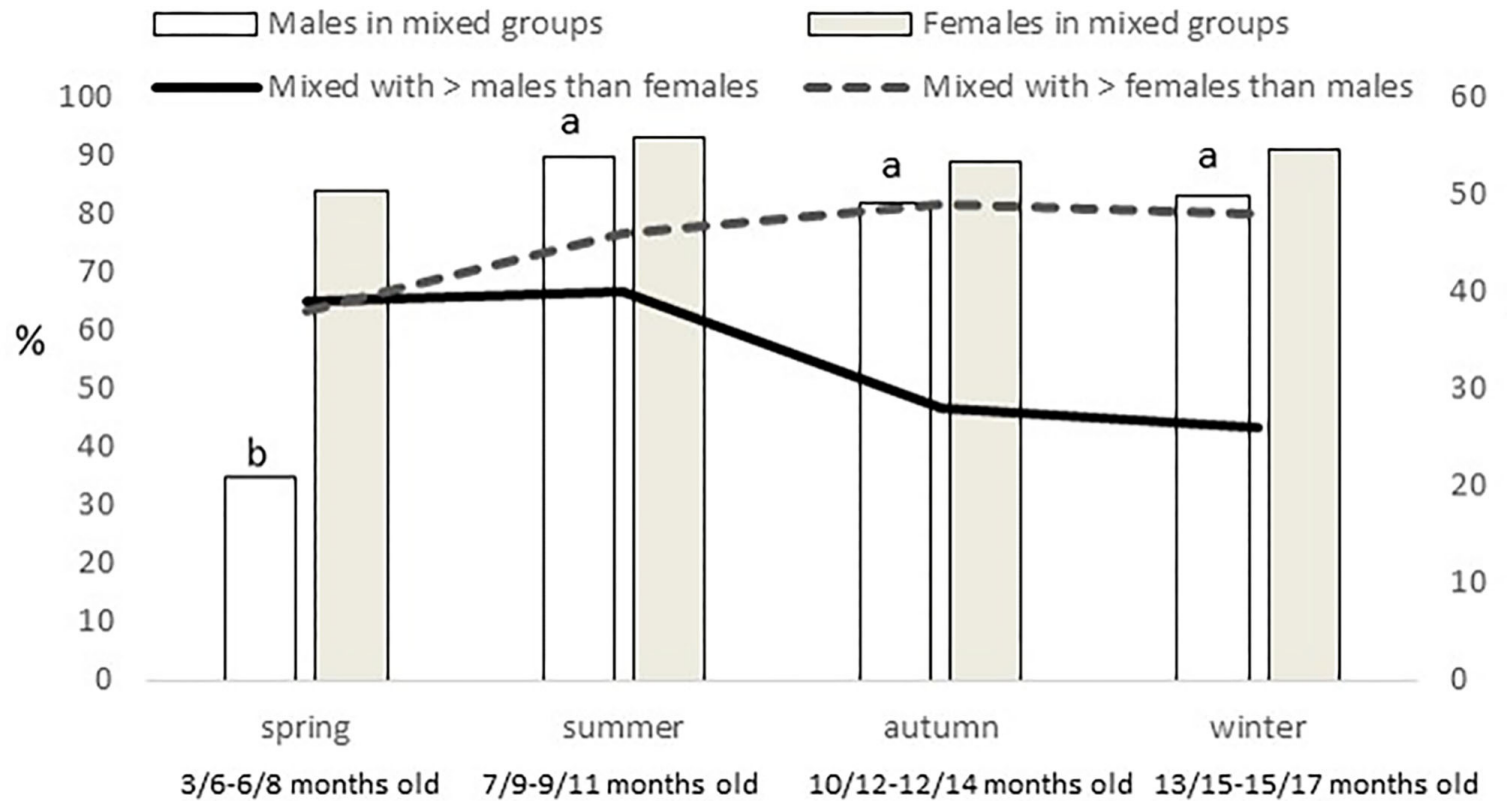
In the left axis and with bars, percentage of males and females out of the total of males and females observed found in mixed groups (groups with at least one male and female). In the right axis and with lines, percentage of mixed groups where more males than females are present and percentage of mixed groups where more females than males are present, observed for the different seasons (spring: March to May; summer: June to August; autumn: September to November and winter: December to February) in free-range Iberian pigs.

Females were more present (*P* = 0.0005) in mixed groups than males. Overall, 91% of the females were found in mixed groups, while this was the case only for 75% of the males. However, these figures were different along the year (*P* < 0.0001; Figure 4). Males were less present in mixed groups in spring than in the rest of the year. Overall, the number of males and females in mixed groups was of 4 ± 0.3 in both cases, but a seasonal effect was found for males (*P* = 0.0059) and females (*P* = 0.0125), in both cases being lower in spring, with 2 ± 0.3 males and 3 ± 0.4 females per mixed group, respectively. Forty-nine percent of the groups had a sex ratio weighted in favor of the females (the maximum proportion being found of 9 females for one male) and another 17% had a sex ratio exactly of one. Therefore, only 34% of the mixed groups had more males than females (the maximum being a proportion of 8.5 males for one female). No effect of season was observed, but an effect of montanera (*P* = 0.0430) was found, with the proportion of mixed groups with more males than females being lower in the montanera (25% out of the total) than in the non-montanera (39% out of the total). The number of females in mixed groups was lower (*P* = 0.0036) during the non-montanera (4.1 ± 0.32) than during the montanera (5.0 ± 0.47). For males, although no significant differences were found, the number of males in mixed groups was 4.3 ± 0.44 and 3.7 ± 0.31, during the non-montanera and the montanera, respectively. Overall, two percent of males and females were found being solitary. However, in males, this percentage was higher (*P* < 0.0001) during the montanera (3.7%) than in the rest of the year (1.9%). Overall, 68% of all solitary animals were males and the rest, 32%, were females.

An interaction distance to the feeder*group type (*P* = 0.0082) and distance to the shelter*group type (*P* = 0.0253) was found for group size, in both cases the male groups found at <10 m from these resources being in bigger groups than female and mixed groups at the same distance, or male groups at other distances. However, more mixed groups were found at <10 m from the feeding area (*P* < 0.0001) than in other positions, while the contrary was found for shelter (*P* = 0.0138) and fences (*P* = 0.0290), where more mixed groups were found to >50 m from these resources than at other distances.

## Discussion

In the present study, only 2% of males and females were found to be solitary along the year. While the benefits of group living have been fully described (11), and being alone can be risky for the individual, it has some advantages as well, such as less competition for resources. Gabor et al. (29) found that 41% of the feral pigs studied were solitary, and Gabor and Hellgren (30) described 27% of the observations as solitary peccaries. In our study, solitary animals accounted for only 9% of the groups, achieving a maximum during the montanera of 11% of the groups (when the animals were older and more space was available), with 3.7% of the males being solitary. In addition, 68% of the solitary animals were males. Therefore, although the percentages are far from those found in feral pigs or peccaries, some sexual divergence in pig behavior during the montanera was found, with males seen more frequently alone.

Mature males are the most seen solitary type of animal in wild boar and warthog (31). Puberty arrives at an average age of 6 to 7 months for females and 7 to 9 months for males (3, 19, 20), and pigs in the present study were studied until 14 to 17 months of age (montanera beginning at 10 to 13 months old). Nevertheless, the males of the present study were castrated, and according to Reiland (32) and Sipos (33) they were just adolescents, as adulthood arrives later than 17 months of age. In consequence, it was not expected to have an important part of the population behaving as mature animals. Accordingly, there is just a minority of the animals being alone, as most of the pigs studied, males and females, formed into groups. Although the risk for predation is low for these Iberian pigs in the areas where they are reared, Martínez-Macipe et al. (34) described in the same farms how animals at the periphery of the groups were more vigilant than were animals in the center, so some anti-predator instinct is maintained in these animals and, accordingly, a high percentage of gregariousness would be expected.

It has been described that pigs in natural environments form matriarchal social groups consisting of 2–4 closely related sows and their offspring of different sizes and ages. As the offspring mature, some of the females split off to form their own group and the males split off to form adolescent bachelor groups, becoming solitary as mature boars (9, 35, 36). Battocchio et al. (37), described a group size of 8–9 animals for wild boars, but Focardi et al. (38) found quite variable group sizes. In fact, Focardi et al. (13) described that group size and the frequency of mixed-sex groups are typically larger in autumn-winter after weaning and during the rut. In production systems, the herds are biased in age (all have the same age) and numbers (it is decided by the farmers and not by the availability of ancestors), so groups are artificially equal in age and big in size. However, in the present study, all of the herd were rarely seen in a single group, and this never happened during the montanera, when the animals were older and more space was available. In contrast, 81% of the groups found in this study had from 1 to 25 individuals, which is in accordance with Romero et al. (39) in peccaries (1–6, 8–14, 16–21) and Gabor et al. (29) in feral pigs (5–27 animals). Rodríguez-Estévez et al. (1), studying an Iberian pig herd with animals of at least 13 months of age during the montanera, found a group mean size of 9 animals, closer to the 12 animals found during the montanera than the 17 during the non-montanera in the present study.

The fact of a reduced number of animals per group during the montanera could be explained because of the age of the animal, because more space was available or because they needed to be more focused on feeding strategies in forest areas and less on forming big groups in more open areas as in an anti-predator strategy (40). In fact, Martínez-Macipe et al. (34) found a clear increase in exploratory/foraging behavior in Iberian pigs during montanera, in comparison to previous seasons where animals were fed, and in the present study, being in the montanera was positively correlated with being closer to trees and negatively with being closer to any other structure (fences, feeding area, drinker, water bath area and fences). Accordingly, Rodríguez-Estévez et al. (1) determined that pigs grazing together accounted for four or fewer individuals, but when they were seen eating acorns there were only one or two animals together. Another effect found during the montanera was an increase in the number of mixed groups that contained more females than males. In fact, the presence of females in mixed groups increased during this period, in comparison to the rest of the year. In males, only a numerical difference was found (not significant), but the combination of both factors is what could explain that during the montanera, only 25% of the mixed groups contained more males than females, being 39% for the rest of the year. As mentioned previously, during the montanera, the number of solitary males increased significantly, and in comparison to other seasons, male group size was lower. Therefore, while females maintained a similar gregariousness in all seasons, males showed more differences along the year, while they were growing and becoming older. During the spring, for instance, these younger males reduced dramatically their presence in mixed groups and very clearly increased the male group size. The consequences of that was a reduction in the mixed group size instead of a reduction in the incidence of these types of groups. In general, females formed smaller groups along the year than did males, explaining this reduced size in mixed group size when males were less interested in staying with females (at the youngest ages).

The figure of little presence of young males in mixed groups just forming big groups without females is typical of several species, such as Przewalsky horses (*Equus przewalskii*), (41), Pyrenean chamois (Rupicapara pyrenaica) (16) or guanacos (*Lama guancioe*) (42), and they are called bachelor groups, described too in wild boar (9, 35, 36). However, it should be expected that this social structure would be maintained longer, and in the present study in summer this changed to another social structure. Actually, summer was the season with more mixed groups found (65%), and males and females reached the maximum involvement in mixed groups (90% of the total of males and 93% of the total of females; Figure 4), and this meant an important change, in comparison to the previous season, again, especially for males. The dehesa is described as an ecosystem in which species of herbs, bushes and trees coexist in a semi-desert regime (43), and summer, when temperatures of 40°C with very low humidity can be reached, is the maximum exponent of this desert-like environment. Iberian pigs can eat *ad libitum* during the montanera (considered the fattening-finishing phase), when several natural resources are available, but during the time before this last phase, restricted feeding is used. In the traditional rearing systems this was due to the scarce feeding resources during the summertime, which is hot and dry. Nowadays, that these animals are supplemented with concentrates, a restricted feeding is deliberately used before fattening to increase fat deposition in the finishing (44). Therefore, although the animals are fed, they are maintained, in most cases, below their needs, and the natural resources in this season are extremely scarce, so this could explain the important changes seen in the group composition and sizes from spring to summer. In fact, during the first year of the present study, the total rainfall registered in the area was of 365 mm (with 60, 0.3, and 240 mm in spring, summer and autumn, respectively) and during the second year of 646 mm (with 266, 6, and 165 mm in spring, summer and autumn, respectively). Therefore, both summers were extremely dry.

In the study area, especially in rainy years, autumn offers another important change in the natural resources available for pigs. In the years when these animals were studied, the temperature ranged from 9 to 32°C and rainfall represented from 25 to 65% of the annual precipitation during autumn. Therefore, although the montanera began approximately at mid-November in most cases (Table 1), autumn, in which pigs are still fed with concentrates for around 80% of the days, should be considered a pre-montanera season. Actually, it is clearly a transitional phase between the hard summer, where animals extremely depend on concentrates that are provided under a restricted regimen to the winter, when a great quantity of acorns will be available and pigs will survive just from natural resources. Accordingly, the groups at <10 m from a tree increased from 59% in summer to the 84% found in autumn (even being under the same landscape conditions, including space allowance). This percentage was then of 94% in winter, but in this case with a clear increase in space allowance (from 7–67 to 0.5–1.8 pigs/hectare). In addition, autumn was the season with the minimum incidence of mixed groups (54%), and an increase in the presence of solitary males and females was observed (Figure 3). In addition, an important reduction from summer to autumn, and maintained in winter, was observed in the number of mixed groups with more males than females (Figure 4), confirming that autumn is really a transitional phase from summer to winter. Later, the main difference found in winter was a slight, but significant, increase of mixed groups in relation to autumn. Therefore, instead of an increase in sexual aggregation, according to the figures found in percentage of males and females in mixed groups (Figure 4), this could be the effect of more mixed groups with a slight reduction in group size. As discussed previously, the differences in group sizes could be associated with the age of the animals, the feeding regimen or space allowance, so animals with a greater presence close to the trees and eating acorns, which happened more frequently in autumn and winter (especially during the montanera) formed smaller groups than in previous periods. Unfortunately, the space allowance, the age of the animals and the feeding regimen are three items associated to the Iberian pig finalized in the montanera, and it is not possible to determine the weight of each one of these factors separately in the results found in the present study.

## Conclusions

The Iberian pigs observed along the year in the present study showed a gregarious behavior, with only 2% of the males and females found as being solitary animals. Although males and females were castrated and spayed, respectively, some divergence in the behavior of both sexes was found. The aggregation structure of females was more stable along the year, with females forming in smaller groups and having a higher presence in mixed groups than did males. Males formed in bachelor groups in spring, when they were the youngest, with a very low presence in mixed groups, but this changed in summer, when the maximum group size and presence of mixed groups was found, which coincides with the hardest season for pigs in terms of availability of food. During the montanera, when the animals were older and more space was available, the presence of males in mixed groups was reduced again and the incidence of solitary males increased. Solitary males represented two-thirds of the solitary animals, males showing less affinity for other males than did females for other females. During the montanera, when the animals were feeding on just natural resources, groups were smaller and were found closer to trees than the rest of the year.

## Data Availability Statement

The raw data supporting the conclusions of this article will be made available by the authors, without undue reservation.

## Ethics Statement

The animal study was reviewed and approved by The Institutional Animal Care and Use Committee (IACUC) of IRTA with the comment No intervention on the animals. Observational study.

## Author Contributions

AD was responsible for the development of the study, supervision of the analysis, collection of the data, and writing the paper. MM-M was responsible for collecting and analyzing the data and writing a first draft of the paper. XM performed advisory functions and collaborated in the development of the study. EM was responsible for training MM-M and collaborated in the supervision of the data analysis and writing of the paper. All authors contributed to the article and approved the submitted version.

## Conflict of Interest

The authors declare that the research was conducted in the absence of any commercial or financial relationships that could be construed as a potential conflict of interest. The reviewer IC declared a past co-authorship with one of the authors XM to the handling editor.
